# Human Nocardia Infections: A Review of Pulmonary Nocardiosis

**DOI:** 10.7759/cureus.304

**Published:** 2015-08-15

**Authors:** Venkataramana Kandi

**Affiliations:** 1 Department of Microbiology, Prathima Institute of Medical Sciences

**Keywords:** human nocardiosis, pulmonary nocardiosis, laboratory diagnosis of nocardia infections

## Abstract

Human nocardiosis may present as an acute or a chronic infection. Although a saprophyte *Nocardia* spp are responsible for superficial skin infections, pulmonary infections, and disseminated nocardiosis usually involving patients who are immunosuppressed and debilitated. Infections in immunocompetent individuals are usually chronic and present non-specific symptoms. Invasive and disseminated nocardiosis is common among patients with weakened cellular immune systems. Clinical diagnosis of pulmonary nocardiosis is difficult owing to the similarity of its presentation with other respiratory pathogens that include *Actinomycetes* members and *Mycobacterium* *tuberculosis*. Laboratory diagnosis of human nocardiosis is plagued by the fact that a culture of *Nocardia* spp requires prolonged incubation periods for isolation which most laboratories fail to follow. The lack of clinical, laboratory, and epidemiological data on the incidence of nocardiosis in humans undermines its significance as a potential pathogen. This review attempts to reexamine the pathogenic potential of *Nocardia* in human infections

## Introduction and background

The bacteria most commonly associated with chest infections include *Haemophilus* *influenzae,* *Klebsiella* *pneumoniae,* and *Streptococcus* *pneumoniae,* and these are the targets of antimicrobials prescribed by a physician when treating chest infections. Chest infections are also caused by other bacterial species that include members of *Actinomycetes*, *Bordetella* *pertussis*, *Mycoplasma* *pneumoniae, *and *Coxiella *spp, which are difficult to cultivate in most laboratories [1-2]. The treatment in the case of respiratory tract infections is usually blind owing to the lack of reliable results from the sputum culture. A sputum culture should not be dismissed as valueless. If carried out correctly, it can be helpful in the management of chest diseases like acute bronchitis, chronic bronchitis, bronchiolitis, bronchiectasis, chronic pulmonary obstructive disorders (COPD), pneumonia, and cystic fibrosis, particularly when they are chronic in nature and recurrent. Using a set of a few enriched, selective and differential media, the organisms present are self-identifying and are reliably isolated. A laboratory report consisting of the identity of the bacterium and its antimicrobial sensitivity pattern can then be issued to the physician. The organisms isolated may be predictable, but with the emergence of resistance to commonly prescribed antibiotics, a specific sensitivity result is essential for guiding appropriate antimicrobial treatment [3-4].

The potential pathogens in cases of chronic chest infections are *Haemophilus* *influenzae* and *Streptococcus* *pneumoniae* followed by *Klebsiella* *pneumoniae*, *Staphylococcus* *aureus* and *Pseudomonas* spp. *Streptococcus* *pneumoniae* is the most common isolate in cases of pneumonia and in cystic fibrosis cases *Pseudomonas* spp and *Staphylococcus* *aureus* are frequent isolates. *Haemophilus* *influenza* is commonly isolated in acute bronchitis and bronchiectasis cases usually in children, young adults and geriatric age patients. Tuberculosis is another most important lower respiratory tract infection affecting lungs and usually causes infections in immunocompromised, debilitated and nutritionally deprived individuals [5]. Other causes for chest infections include microorganisms of fungal and viral origins. *Aspergillus* spp (mostly *A flavus and A fumigatus*), *Candida* spp, and *Cryptococcus* spp cause pulmonary infections usually in immunocompromised patients, which include HIV/AIDS (human immunodeficiency virus infection/acquired immunodeficiency syndrome) patients [6-7]. Most viral respiratory tract infections predispose individuals to secondary bacterial or fungal infections necessitating antimicrobial therapy.

## Review

*Nocardia* spp belong to the aerobic actinomycetes group (Phylum: Actinobacteria, Order: Actinomycetales) of bacteria which are gram-positive bacilli showing branching filamentous forms, are non-spore forming, and mildly acid-fast bacteria [1]. These bacteria are saprophytic and are found in soil and water [2]. Among more than 85 identified species of *Nocardia, *approximately 25 species are associated with human infections and include *Nocardia asteroides* complex (more than 50% human cases), *N. brasiliensis, N. abscessus*, *N. cyriacigeorgica, N. farcinica, N. nova,* *N. transvalensis complex, N. nova* complex,* N. pseudobrasiliensis, *and the recently reported *Nocardia veteran and N. cerradoensis* [8-9]*. *Human infection with *Nocardia* can result due to inhalation (pulmonary nocardiosis-pneumonia, lung abscess, and cavitary lesions) or contact with the bacteria via a cut or abraded skin (cutaneous nocardiosis-cellulitis, ulcers), and the infection can then disseminate to the brain, kidneys, joints, heart, eyes, and bones [10-13]. *Nocardia* spp may also result from hospital-acquired infections, usually involving catheterized patients and those who undergo surgeries (postoperative infections) [14]. Human-to-human transmission is not documented. Pulmonary infection with *Nocardia* spp show clinical symptoms similar to those suspected with pulmonary tuberculosis (fever, cough, chest pain, night sweats, weight loss and pneumonia) [15]. Infections with *Nocardia* spp usually occur in individuals with weakened immune system and can include patients suffering from diabetes, malignancies, HIV/AIDS, lung disorders like pulmonary alveolar proteinosis (plugged lung air sacs), individuals with connective tissue disorders, chronic alcoholism, transplant patients, and patients on corticosteroid therapy. In developing countries, including the United States of America, it has been noted that more than 60% of human nocardiosis occurs in immunocompromised individuals and that males are more prone to infection than females (3:1) [8, 16-21]. The data on human nocardiosis is limited in the literature and is usually present as isolated case reports undermining its significance in human infection [22-24]. Persistent and disseminated human infection with *Nocardia cyriacigeorgica* in an otherwise immunocompetent individual was reported recently in the literature which was treated employing a strategic combination therapy that lasted for more than a year. It has been noted that a delay in diagnosis among immunosuppressed patients infected with *Nocardia* may be responsible for treatment failure and poor prognosis [25]. Another report which reviewed the history of human nocardiosis has revealed that clinicians should suspect infection with *Nocardia* and notify the same to the laboratory so that the necessary steps are taken by laboratory specialists to isolate, identify, and sensitivity pattern of the *Nocardia* spp [26]. Co-infection with *Nocardia* spp in patients suffering from mycobacterial lung infection highlight the importance of laboratory diagnosis that may facilitate better patient management [27]. A previous study has also elaborated on the types of radiological findings in pulmonary nocardiosis that include consolidation of the lungs, presence of nodules and masses, pleural effusion, and extension of lung infection towards the chest wall resulting in abscess [28]. A very recent research report has elaborated on the significance of ecological and epidemiological studies on the occurrence of pathogenic *Nocardia* spp in the soil and environment [29].        

### Laboratory identification of Nocardia spp

**Figure 1:**
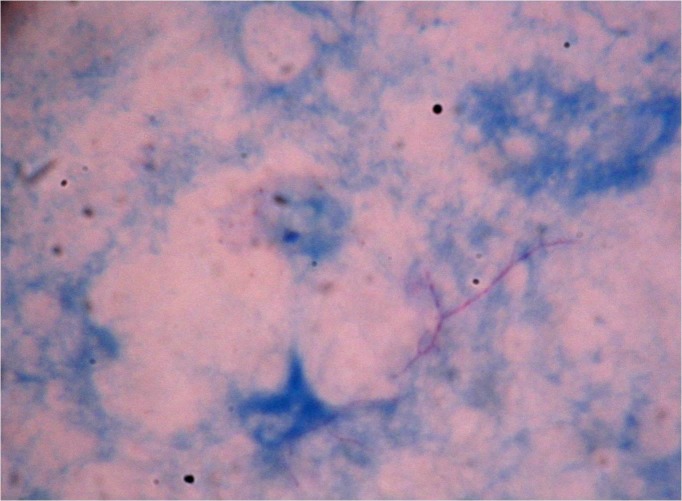
Pink colored filamentous and branched bacilli appearing in modified acid fast stained smear of respiratory secretions

Laboratory diagnosis of human nocardiosis includes microscopy and culture. Identification of *Nocardia* is more rapid, precise, and accurate with polymerase chain reaction (PCR) and 16S rDNA sequencing than with conventional phenotypic methods, which include microscopic, cultural, and biochemical properties [30-32]. Modified acid-fast staining using 1% sulphuric acid as a decolorizer is employed to identify *Nocardia* microscopically in clinical samples, where pink colored filamentous branching bacilli are observed as shown in Figure 1.

*N. asteroides* grows well on Lowenstein-Jensen’s medium (LJ medium) when incubated between 30^0^C and 37^0^C. *Nocardia* spp also grow readily on blood agar and Saboraud’s dextrose agar (SDA). Agar medium enriched with yeast extract (buffered charcoal yeast extract agar (BCYEA)) may improve the chances of isolation from clinical specimens. Colonies of *Nocardia* spp usually appear after 48 hours of incubation and a visible growth may take more than one week for some species. Laboratories may fail to isolate *Nocardia* from clinical specimens if plates are discarded after 48 hours, as regularly followed in most parts of the world. Application of molecular methods is recommended to identify the suspected human nocardiosis using multi-locus sequence analysis (MLSA) to reduce underreporting and misdiagnosis and is important for clinical and epidemiological purposes [33]. A recent report from Brazil has noted 0.12% of the sputum samples collected from patients suspected to be suffering from infection with *Mycobacterium* *tuberculosis* (Pulmonary tuberculosis) have grown *Nocardia* in Lowenstein-Jensen’s medium. This study has also elaborated on the significance of a presumptive clinical diagnosis in the improvement of laboratory isolation of *Nocardia* spp from clinical samples [34].

### Antimicrobial chemotherapy in nocardiosis

Although *Nocardia* spp are susceptible to most of the penicillin and cephalosporin group of antibiotics, the cause of concern is the probability of the emergence of antimicrobial resistance among some species [8, 35]. Antibiotics effective against gram-positive bacteria, including linezolid, ampicillin, erythromycin, and minocycline are used to treat nocardiosis. Considering the emergence of antibiotic resistance a combination of sulfonamide (trimethoprim/ sulfamethoxazole), ceftriaxone and amikacin may be preferred to treat human nocardiosis [36]. A recent paper has highlighted the significance of the organ involved, its antimicrobial susceptibility pattern, and the use of combination antibiotic therapy in the treatment of human nocardiosis [37]. A ten-year retrospective study of antibiotic susceptibility patterns of *Nocardia* spp isolated from the United States of America revealed that more than 50% isolates were resistant to trimethoprim/sulfamethoxazole (TMP/SMX). This study has also recommended that a combination therapy including TMP/SMX, ceftriaxone and imipenem can be initiated until the sensitivity report is available [38].

## Conclusions

Clinicians, chest disease specialists, and clinical microbiologists should consider carefully the possibility of human nocardiosis. Clinical microbiology laboratories must follow standard protocols when performing sputum cultures and consider isolation and identification of *Nocardia* spp from various clinical specimens for better patient management in case of chest infections.
